# Exposure to blue light reduces antimicrobial resistant *Pseudomonas aeruginosa* isolated from dog ear infections

**DOI:** 10.3389/fmicb.2024.1414412

**Published:** 2024-07-04

**Authors:** Adriano M. Gigante, Mohammad A. Hadis, Bailey Secker, Stephen C. Shaw, Paul R. Cooper, William M. Palin, Michael R. Milward, Robert J. Atterbury

**Affiliations:** ^1^School of Veterinary Medicine and Science, University of Nottingham, Leicestershire, United Kingdom; ^2^School of Dentistry, University of Birmingham, Birmingham, United Kingdom; ^3^Department of Oral Sciences, Faculty of Dentistry, University of Otago, Dunedin, New Zealand

**Keywords:** otitis externa, blue light, *Pseudomonas aeruginosa*, enrofloxacin, AMR (antimicrobial resistance), canine

## Abstract

**Introduction:**

*Pseudomonas aeruginosa* is a leading cause of canine otitis externa. Enrofloxacin is often applied topically to treat this condition, although recalcitrant and recurring infections are common. There is evidence that exposure to blue light (400–470 nm) has a bactericidal effect on *P. aeruginosa* and other microorganisms.

**Methods:**

In the present study, we tested the biocidal effect of blue light (375–450 nm), alone or in combination with enrofloxacin, against six isolates of *P. aeruginosa* from dogs with otitis externa (5 of which were resistant to enrofloxacin).

**Results:**

Treatment of planktonic cell cultures with blue light resulted in significant (*p* < 0.5) reductions in Colony Forming Units (CFU) for all seven strains tested, in some cases below the limit of detection. The greatest bactericidal effect was observed following exposure to light at 405 nm wavelength (*p* < 0.05). Exposure to blue light for 20 min usually resulted in a greater reduction in *Pseudomonas aeruginosa* than enrofloxacin treatment, and combination treatment typically resulted in the largest reductions in CFU. Analysis of the genome sequences of these strains established that enrofloxacin resistance was likely the result of a S466F substitution in GyrB. However, there was no clear association between genotype and susceptibility to blue light treatment.

**Discussion:**

These results suggest that blue light treatment, particularly at 405 nm wavelength, and especially in combination with enrofloxacin therapy, could be an effective treatment for otherwise recalcitrant canine otitis externa caused by *Pseudomonas aeruginosa*. It may also provide a way of extending the usefulness of enrofloxacin therapy which would otherwise be ineffective as a sole therapeutic agent.

## Introduction

Otitis externa (OE) is inflammation of the external ear canal (Bajwa, 2019) and is one of the most common dermatological conditions in canines, affecting up to 20% of dogs worldwide (Hill et al., 2006), and was the second most common diagnosis for dogs in the UK in 2016 (O’Neill et al., 2021). Canine OE is a multifactorial disease, consisting of primary disease complicated by secondary infection, perpetuating tissue changes, and predisposing factors that increase the risk of disease such as ear canal anatomy or excessive wetting. A primary factor, commonly allergy, causes the initial inflammation in the ear canal leading to dysbiosis, overgrowth and then increasingly severe secondary infections. The resultant tissue changes to the ear canal prevent the resolution of OE and are known as perpetuating factors (Secker et al., 2023).

*P. aeruginosa* is a significant cause of recalcitrant and recurrent canine OE (Zamankhan Malayeri et al., 2010; Bajwa, 2019), owing to its intrinsic reduced sensitivity to many antimicrobials, which is partly attributed to its ability to form biofilms (Chan et al., 2019; Moyaert et al., 2019). Fluoroquinolones, like enrofloxacin (EFX), are topically applied to treat OE in dogs frequently as their function is not impaired by the presence of pus unlike other antibiotic classes (Nuttall, 2016). While up to 67.7% of clinical isolates of *P. aeruginosa* from OE exhibit resistance to EFX (Bourély et al., 2019) the high concentration of enrofloxacin used by this route may overcome the resistance described by traditional microbiological methods that assume systemic therapy.

**FIGURE 1:**
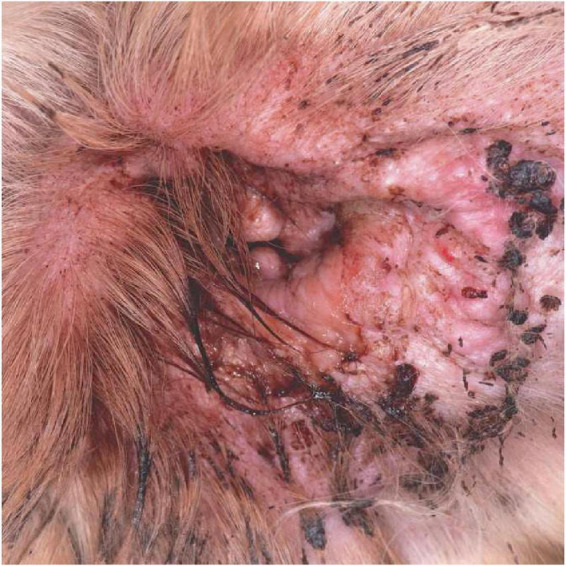
The clinical appearance of a severe otitis externa with *Pseudomonas* infection. Note the erythematous swollen canal opening with marked tissue change and a marked purulent discharge.

Antimicrobial resistance (AMR) and multidrug resistance (MDR) are common in *P. aeruginosa* isolates from canine OE, despite improved antibiotic prescribing practices (Bourély et al., 2019; Petrov et al., 2019), and this plays a major role in treatment failure. In dogs with any ear disease there is considerable pain and pruritus (Figure 1), but the intractable nature of *Pseudomonas* otitis is such that dogs will often require surgical removal of the external ear canal and partial removal of middle ear resulting in almost complete hearing loss in a procedure termed total ear canal ablation and bulla osteotomy (TECABO). In a small number of cases post-operative *Pseudomonas* infection remains problematic (Smeak, 2016). The difficulties of treatment demand new and more effective remedies for this condition. Several studies have used alternative approaches to reduce *Pseudomonas aeruginosa* by 4–5 log units both *in vitro* and in a mouse infection model by employing antimicrobial peptides (Murphy et al., 2024) and bacteriophage endolysins (Briers et al., 2011; Raz et al., 2019).

Light therapy has been rarely used in veterinary medicine, but recently has attracted more attention due to the growing need for alternatives to antibiotics. A commercial product using light to activate photoactive gels applied to the skin is used in small animals in the UK, termed fluorescence biomodulation, and has roles in treating deep and superficial skin infections, likely through an anti-inflammatory effect (Marchegiani et al., 2021, 2023). Similarly, low-level laser therapy has been reported as useful as an adjunctive therapy in acral-lick dermatitis with both anti-staphylococcal and anti-inflammatory effects suggested (Schnedeker et al., 2021).

Antimicrobial blue light (aBL) is a non-antibiotic approach to inactivate microorganisms (Wang et al., 2017b). Blue light is safer to use than UV light, and in the 400–470 nm wavelength range, has been used experimentally to inactivate Gram-negative pathogens (Wang et al., 2016) as well as planktonic Gram-positive bacteria, mycobacteria, molds, yeasts and dermatophytes (Wang et al., 2017b). aBL is thought to act by generating reactive oxygen species (ROS) upon excitation of endogenous chromophores, such as porphyrins (Schmid et al., 2019) and flavins (Makdoumi et al., 2019), within microbial cells. ROS, including singlet oxygen and superoxide anions, can cause oxidative damage to essential biomolecules within the microbial cells, ultimately resulting in cell death. This mechanism of action differs from that seen with UV light as bacterial chromophores have absorption peaks in the range of 390–425 nm which are weakly absorbed by eukaryotic cells. Furthermore, small amounts of ROS produced by blue activation in eukaryotic cells can be beneficial in promoting tissue repair (Mohamad et al., 2022). The specific mechanisms of aBL may vary depending on the microbial species, the endogenous chromophores present within the cells, and the presence of endogenous antioxidants, such as staphyloxanthin, which may protect bacteria from ROS attack (Leanse et al., 2021). Moreover, the photolysis of these antioxidants by aBL may sensitize bacteria to oxidative stress and antimicrobial agents. The antimicrobial action of aBL has been reviewed in detail elsewhere (Leanse et al., 2022).

There is some evidence of a synergistic effect between aBL and antimicrobials (Leanse et al., 2020a), which may extend to both antibacterial and antifungal activity (Dai and Hamblin, 2017; Leanse et al., 2020b). Unlike antimicrobial therapy, there is no evidence of increasing tolerance to aBL treatment among multiple microorganisms, including *P. aeruginosa* (Amin et al., 2016), as reviewed elsewhere (Wang et al., 2016).

In this study, six clinical strains of *P. aeruginosa* isolated from dogs with OE were exposed *in vitro* to aBL at specific wavelengths between 375 and 450 nm, with or without EFX. The effect of the treatment was assessed by CFU/mL reduction at 24 h after a treatment of 20 min. The genomes of these *P. aeruginosa* strains were assessed for AMR and virulence determinants, as well as diversity. We also assessed whether there were genomic markers associated with aBL sensitivity.

## Materials and methods

### Light device development

**FIGURE 2:**
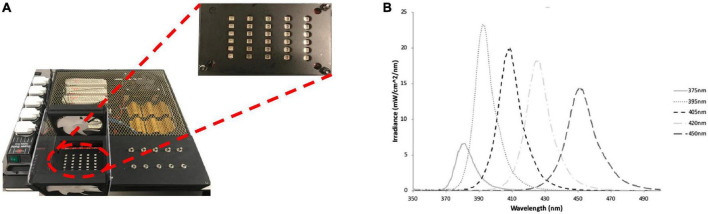
**(A)** The LED array used to irradiate bacteria cultures with plate showing channel 375–450 nm (left to right) and **(B)** the spectral irradiance for each channel, respectively. Figure originally published in (Halstead et al., 2019), reproduced with the authors’ permission.

A multi-wavelength array (MWA) was designed and manufactured “in-house” based on previous work by the authors (Hadis et al., 2017; Halstead et al., 2019), for use in the high-throughput analysis of the antibacterial effects of five specific wavelength bands of aBL with peak wavelengths of 375, 395, 405, 420, and 450 nm (Figure 2 and Supplementary Table 1). The array was designed to intimately fit with 96-well cell culture plates and each wavelength was delivered by surface mounted diodes and optical lenses. A fiber-based UV-Vis spectrometer (USB4000, Ocean Optics, UK) which comprised a 200 μm optical fiber and an opaline glass CC3 cosine corrector (3.90 mm diameter of collection area; 6.35 mm outer diameter; Ocean Optics, UK) and calibrated in-house to NIST standards against a traceable light source (Mikropack DH2000/ Ocean Optics, UK) was used to assess the absolute spectral irradiance for each LED. A black 96-well plate (Corning, Sigma Aldrich) was placed into the array and the irradiance delivered to the base of the plate was measured. The MWA was subsequently calibrated to deliver an irradiance of approximately 98 mW/cm^2^ (375 nm), or 338–372 mW/cm^2^ (all other wavelengths) at the base of 96-well plates (Table 1). Full characterization of a similar MWA array, which includes, temperature and beam profile has previously been reported by the authors (Halstead et al., 2019).

**TABLE 1:** Association of enrofloxacin resistance with amino acid substitutions.

Strain	GyrA	GyrB	ParC	ParE
2943	D652Y	S466F		
29758	D652Y	S466F		
29878-1	D652Y	S466F		
464429				
467523	D652Y			
488402	T847A, S912_E913del			

Amino acid substitutions and deletions of *gyrA*, *gyrB*, *parC*, and *parE* from genome sequences of six *P. aeruginosa* from cases of canine OE measured by alignment with the amino acid sequence from PAO1.

### Bacteria strains and culture conditions

The *P. aeruginosa* clinical strains used in this study were isolated from dogs with canine OE in the UK (464429, 467523 and 488402) and Denmark (2943, 29758 and 29878-1). The isolates were confirmed by characteristic growth on Cetrimide agar, and testing positive for oxidase activity and by 16S rRNA PCR. Following confirmation, *Pseudomonas* isolates bacteria were subsequently cultured in 20 mL Luria-Bertani (LB) medium (Oxoid™) in a sterile 30 mL universal tube at 37°C overnight (18 h) with shaking (150 rpm/min).

### Enrofloxacin susceptibility

Susceptibility of the *P. aeruginosa* strains to EFX was determined using the disk diffusion method described by the European Committee on Antimicrobial Susceptibility Testing (EUCAST, 2024). Briefly, a single colony from an overnight culture of each bacterium on Mueller-Hinton agar (Oxoid) was suspended in saline to achieve an OD_600_ of 0.1. A sterile cotton-tip swab was then soaked in this suspension and used to inoculate a Petri dish containing Mueller-Hinton agar. An EFX loaded disk (0.5 μg, Oxoid) was applied on the surface of the agar and the Petri dish was then incubated for 18 h at 37°C before measuring zones of inhibition using digital calipers. For minimum inhibitory concentration (MIC), the EUCAST method was used, following ISO 20776-2 using EFX concentrations ranging from 73 to 0.036 mg/L. Plates were examined for growth after 18 h incubation. As EUCAST does not have specific breakpoint data for EFX, ciprofloxacin breakpoints were used as a fluoroquinolone representative. In addition, the Clinical and Laboratory Standards Institute (CLSI) veterinary guidelines (CLSI, 2018) were used as a comparison as they do provide EFX breakpoints.

### Submerged biofilm assay

U-bottom polystyrene 96-well microtiter plates (Greiner Bio-One) were used to assess the biofilm forming ability of the clinical *P. aeruginosa* isolates as previously described, with some modifications (Coffey and Anderson, 2014). Briefly, overnight cultures of *Pseudomonas* in LB broth were diluted 1:100 in fresh LB broth and 100 μL aliquots were transferred to the 96-well plate in triplicate, followed by static incubation at 37°C for 24 h. Planktonic cells were removed following incubation, and the wells were washed with 125 μL of Ca-HEPES buffer before being stained with 0.1% (v/v) crystal violet solution. After removing excess crystal violet, the wells were de-stained with 100% ethanol, and a Tecan GENios Pro was used to record absorbance (595 nm). Isolates were then classified as either strong, moderate, weak, or non-biofilm producing as previously described (Stepanović et al., 2000).

### *In-vitro* irradiation assay

A single colony of the *P. aeruginosa* strain to be tested was used to inoculate 15 mL of LB medium which was incubated in a sterile 35 mL tube at 37°C overnight (18 h) with shaking (150 rpm/min). Following incubation, cells in a 10 mL aliquot of the culture were pelleted (10 min, 5,000 *g*), washed twice and resuspended in 10 mL phosphate buffered saline (PBS). The OD_600_ was adjusted to 0.1 then diluted 100-fold in PBS to obtain the working culture dilution. Each well of a 96-well imaging plate (Corning) was inoculated with 135 μL of diluted bacterial culture and 15 μL of EFX stock suspension (22.5 mg/mL in PBS) or 15 μL of PBS. The plate was immediately placed in the light emitting device and irradiated for 20 min. The aBL wavelengths used were 375, 395, 405, 420 and 435 nm. Following irradiation, the contents of each well were decimally diluted, and 10 μL volumes of each dilution spotted onto the surface on an LB agar plate in triplicate. The plates were incubated at 37°C for 18 h before enumeration of colonies.

### *Pseudomonas* genome sequencing

Whole genome sequencing was performed by MicrobesNG (Birmingham, UK) using short reads (Illumina MiSeq) and long reads (Oxford Nanopore). The quality of the Illumina paired-end reads was initially assessed using FastQC v0.11.8 (Andrews, 2010) before trimming adapters and poor-quality reads using FastP v0.12.4 (Chen et al., 2018). Subsequently, Flye v2.9.2-b1786 (Kolmogorov et al., 2019) was used for *de novo* assembly. The resulting assemblies were processed using Circlator v1.5.5 (Hunt et al., 2015) and Bandage v0.8.1 (Wick et al., 2015) to assess integrity. One round of long read polishing was performed using Medaka v1.11.1 (ONT, 2023) followed by two rounds of short read polishing, first with Polypolish v0.5.0 (Wick and Holt, 2022) and then POLCA from the MaSuRCA toolkit v4.0.9 (Zimin and Salzberg, 2020), both with default settings. Finally, genomes were reoriented to begin with *dnaA* using dnaapler v0.4.0 (Bouras et al., 2024) and annotated using Bakta v1.8.2 (Schwengers et al., 2021). The genome sequences derived in this study can be accessed from GenBank under the Bioproject accession number PRJNA1078132.

### Bioinformatic analysis

*Pseudomonas* strains were allocated to multilocus sequence types (MLST) *in silico* using mlst v2.23.0 (Seemann, 2023) and the PubMLST database (Jolley et al., 2018). A neighbor joining phylogenetic tree was constructed using Mashtree v1.4.6 (Katz et al., 2019) and visualized using MEGA v11.0.13 (Tamura et al., 2021). The percentage identity of biofilm associated genes was assessed with a custom database using gene sequences from *P. aeruginosa* PAO1 (NC_002516.2), acquired from NCBI using ABRicate v1.0.1 (Seemann, 2015). A complete list of the genes can be found in Supplementary Table 2 and includes genes located within the *psl*, *pel* and alginate (*alg*) operons in addition to genes previously reported to be associated with biofilm formation (Franklin et al., 2011; Kiel et al., 2022). Screening of antimicrobial resistance genes was performed using ABRicate (Seemann, 2015) with the CARD (Alcock et al., 2023) database. Subsequently, the presence of amino acid substitutions and deletions was investigated for five genes, *gyrA*, *gyrB*, *parC*, *parE*, and *mexR*, by comparing the sequences to the sequences of PAO1 (NC_002516.2) using the ClustalW algorithm in MEGA v11.0.13 (Tamura et al., 2021).

### Statistical analysis

Bacterial counts were log_10_-transformed prior to analysis. Differences in the CFU counts from aBL/EFX treated and untreated cultures were determined using the Kruskal-Wallis test, followed by Dunn’s multiple comparison test. All analysis was performed using Graphpad Prism 10.1 (GraphPad Inc.).

## Results

### Enrofloxacin sensitivity and biofilm formation

The sensitivity of six clinical *P. aeruginosa* strains to EFX was determined using disk diffusion (Supplementary Figure 1). Using the EUCAST ciprofloxacin breakpoints, all of the clinical *P. aeruginosa* isolates in addition to *P. aeruginosa* PAO1 were resistant to enrofloxacin. Comparatively, all but one of the strains (464429, which displayed intermediate sensitivity), were resistant based on CLSI criteria. The MIC results obtained by micro-dilution (Supplementary Table 3) demonstrate all strains can be considered resistant to EFX (MIC > 4 mg/L) except 464429 that falls under the category of intermediate resistance (> 0.5 and < 4 mg/L), which agrees with the disk diffusion diameter interpretation. The five enrofloxacin-resistant strains, along with *P. aeruginosa* PAO1, were also characterized as strong biofilm producers, with 464429 producing no quantifiable level of biofilm (Supplementary Figure 2). When the genomes of the clinical *P. aeruginosa* isolates were assessed for the presence of 53 genes that are associated with biofilm formation; *pslA*, *pslB*, *pslC*, and *pslD*–associated with Psl exopolysaccharide production—were absent in strain 464429 (Supplementary Figure 3 and Supplementary Table 2).

### Identification of genomic determinants of EFX resistance

**FIGURE 3:**
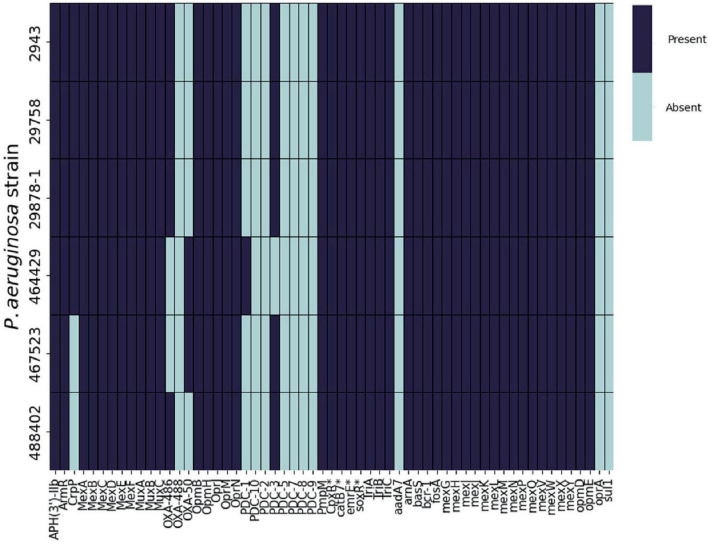
Presence or absence of AMR genes in *P. aeruginosa* clinical isolates. ABRicate was used in combination with the Comprehensive Antibiotic Resistance Database (CARD) to determine the presence of antibiotic resistance genes in the genomes of six clinical *P. aeruginosa* isolated from canine otitis externa. *CARD identified these products specifically belonging to *P. aeruginosa*.

In order to further investigate the basis of EFX resistance in the *Pseudomonas* strains used in this study, the genomes of the clinical isolates were screened for the presence of known antimicrobial resistance (AMR) genes using the CARD database (Figure 3).

The repertoire of resistance genes was similar across all of the strains. However, following analysis of the amino acid sequences of *gyrA*, *gyrB*, *parC*, *parE*, and *mexR* (Table 1), mutations in some of these genes were previously associated with EFX resistance in *P. aeruginosa* isolated from dogs (Park et al., 2020). Four strains (2943, 29878-1, 29758 and 467523) possessed a single substitution in GyrA, Asp652Tyr, in addition to strain 488402 which has a single substitution Thr847Ala. Regarding GyrB, three strains (2943, 29878-1 and 29758) show a single substitution of Ser466Phe. No mutations were found on *mexR, parC*, or *parE* genes for any of the six strains when aligned with PAO1. It is also noteworthy that no mutations were identified in *P. aeruginosa* 464429.

### Blue light treatment reduces *P. aeruginosa* viable counts

**FIGURE 4:**
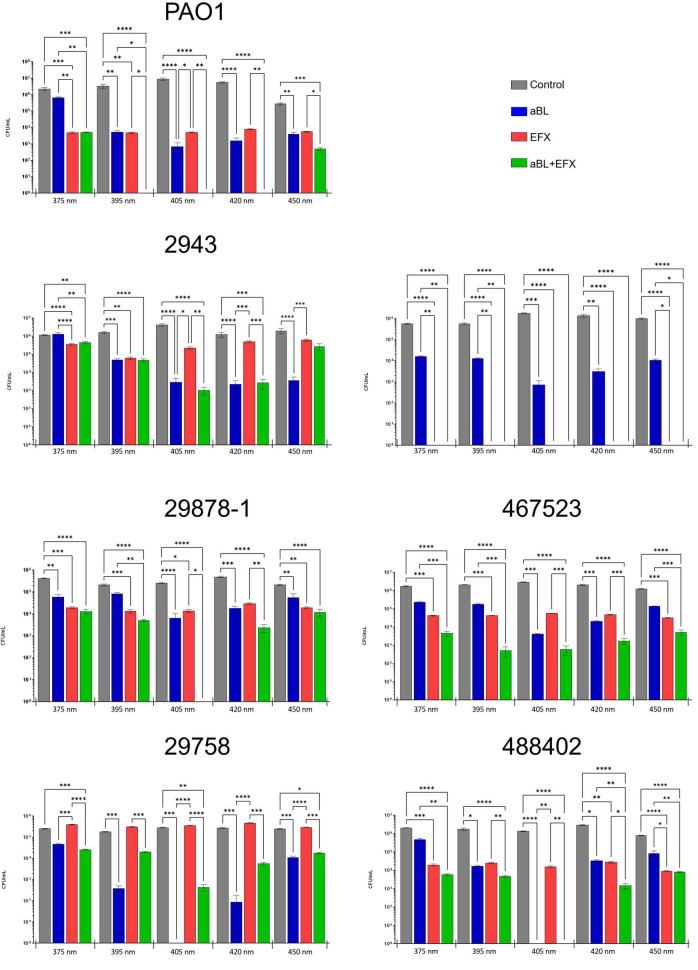
Effect of aBL and EFX alone or in combination on *P. aeruginosa* clinical isolates and PAO1 strains. Cultures of *P. aeruginosa* were exposed to different wavelengths of blue light (*x*-axis) alone, or in combination with enrofloxacin treatment. Separate cultures were exposed to blue light alone (blue), enrofloxacin alone (red), both treatments (green) or no treatment (gray). The values represent log_10_ CFU/mL ± SEM based on three biological and six technical replicates. The absence of a bar represents counts below the limit of detection (10^2^ CFU/mL).

The effect of aBL (with or without enrofloxacin) on the viability of *P. aeruginosa* strains is presented in Figure 4. In almost all cases the use of aBL alone was associated with a decrease in viable counts across all wavelengths tested, and for all strains. Overall, the greatest reductions in viable counts were recorded following treatment with the 405 nm light (*p* ≤ 0.001) when compared to the other wavelengths tested. At 405 nm, strain 488402 showed the greatest reduction in counts (≥ 4.34 log_10_ CFU/mL) and strain 29878-1 showed the lowest counts reduction (1.77 log_10_ CFU/mL). Significant reductions (*p* ≤ 0.001) of close to 3 log_10_ CFU/mL were recorded in four of the strains tested (PAO-1, 2943, 29758, 464429) following treatment with 420 nm light. Significant (*p* ≤ 0.01), but more variable reductions, were obtained after exposure to 395 nm light, where reductions of up to 2.97 log_10_ CFU/mL recorded for three of the strains (POA-1, 29758 and 488402). The least effective treatment was for 375 nm light, where a significant reduction was recorded for one strain only 1.19 log_10_ CFU/mL (29878-1). Noteworthy, treatment with the most effective wavelength of light (405 nm) was associated with reductions below detectable limits (10^2^ CFU/mL) in two of the tested strains (29758 and 488402).

### Combination of EFX and aBL can further reduce *Pseudomonas* viability

All *P. aeruginosa* isolates used in this study were obtained from refractory OE infections in dogs. As such, these strains were expected to exhibit a reduced sensitivity to EFX treatment. This was indeed the case for all but one of the clinical isolates (464429) which appeared to be sensitive to clinical concentrations of EFX, despite exhibiting intermediate sensitivity using a disk diffusion assay. When compared to untreated control cultures, EFX decreased viable CFU/mL counts for strains PAO-1 (2.89 log_10_), 2943 (1.46 log_10_), 29878-1 (1.48 log_10_), 464429 (up to 3.97 log_10_), 467523 (1.83 log_10_) and 488402 (2.02 log_10_), while no significant reduction was recorded for strain 29758.

The effect of combining EFX and aBL varied according to the wavelength of aBL used. Noteworthy, at 405 nm, the combination treatment reduced viable counts for six out of seven strains tested. The counts were reduced below detectable limits in four strains (PAO-1, 464429, 29878-1 and 488402), which was equivalent to a reduction of ≥ 5.25 log_10_ CFU/mL. One strain (2943) showed a small, further reduction in counts of 3.48 log_10_ CFU/mL following combination treatment (compared with 3.05 log_10_ CFU/mL for aBL alone). The effect of combination treatment on strain 488402 could not be determined as counts were below detectable limits for aBL treatment with or without EFX. Interestingly, combination treatment resulted in higher counts for strain 29758 than aBL used alone. However, overall, most counts following combination treatment were significantly lower than either treatment used alone. It is noteworthy that the combination treatment reduced the numbers to below detectable limits for PAO-1 (at 405 and 420 nm) and 29878-1 (at 405 nm)–a much greater decrease then either treatment independently–suggesting the possibility of either a synergistic or additive effect.

### Multilocus sequencing type of *P. aeruginosa* offered no predictive value on aBL treatment outcome

None of the strains from Denmark could be allocated to a known ST, whereas UK strains 488402, 467523 and 484919 were allocated to ST 557, 3014 and 111, respectively (Table 2). PAO1 was assigned to ST 549, and ATCC 27853 to ST 155, which concurs with previous reports (Winsor et al., 2016; Jolley et al., 2018s).

**TABLE 2:** Seven multilocus sequence typing genes of six *P. aeruginosa* clinical isolates tested.

Strain	ST	*acsA*	*aroE*	*guaA*	*mutL*	*nuoD*	*ppsA*	*trpE*
ATCC 27853	155	28	5	36	3	3	13	7
PAO1	549	7	5	12	3	4	1	7
488402	557	11	5	12	11	4	4	20
467523	3014	16	5	12	3	3	1	18
2943 29758 29878-1	Unknown	16	5	30	72	4	13	7,321
464429	111	17	5	5	4	4	4	3

PAO1 plus ATCC 27853 strains were included as a reference strain with and without resistance to fluoroquinolones to serve as a comparative landmark to the clinical isolates. The six isolates fall under three different ST groups and an uncategorised group. ST, sequence type.

**FIGURE 5:**
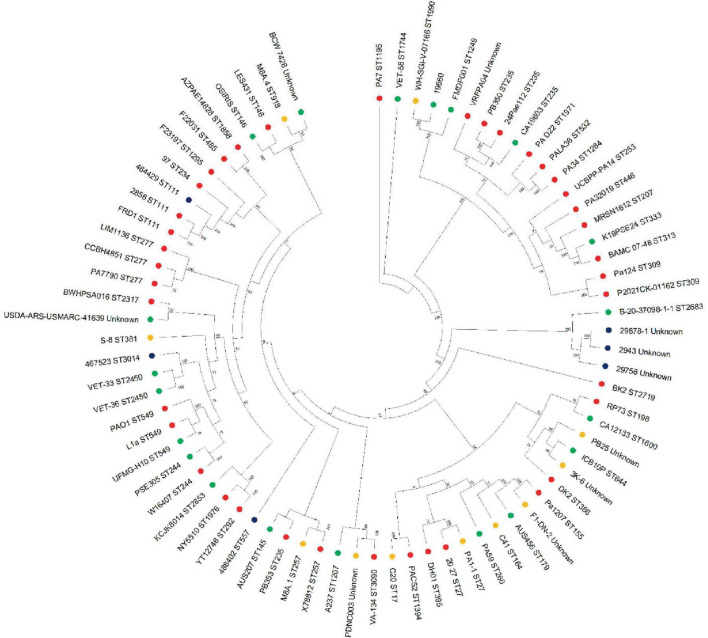
Phylogenetic tree of *P. aeruginosa* strains. Mashtree was used to calculate the phylogenetic distances of the *P. aeruginosa* isolates from this work in addition to 70 isolates from other sources to create a neighbor joining tree which was visualized using MEGA. Strains isolated from the current study (blue), human infections (red), animal infections (green) and environmental sources (gold) were included in the comparison.

Whole genome sequences of these strains, along with 70 *P. aeruginosa* strains from human, animal and environmental sources were used to construct a phylogenetic tree (Figure 5) and resolve the similarity between strains which were untypable by MLST. This revealed that the Danish strains clustered together on the same branch with another isolate from an animal infection (B-20-37098-1-1, ST 2683). The phylogenetic tree identified three main branches, with all of the isolates from this study clustering in the largest group, which also contained PAO1. Interestingly, the canine isolates from this study clustered with isolates from other sources (including non-clinical), which is consistent with our current understanding of the non-clonal nature of *Pseudomonas* from canine OE (Secker et al., 2023).

## Discussion

In this study, we investigated the potential effect of aBL treatment for killing *P. aeruginosa* isolated from dogs with OE. The results showed that viable counts of *Pseudomonas* were reduced following exposure to aBL, with or without the use of EFX. For all strains tested, the reduction in CFU/mL is most pronounced at 405 nm, in some cases surpassing the reduction observed when using EFX alone. This is significant given that the concentration of EFX used in this study was equivalent to that used in clinical treatment (Metry et al., 2012). The combination of aBL with EFX typically resulted in a greater reduction in viable counts for most of the wavelengths tested, in some cases reducing numbers below detectable limits. This result suggests, for the first time, that aBL might be a useful adjunct to antimicrobial treatment currently available at veterinary practices, and could augment the efficacy of EFX chemotherapy.

Further work will be required to develop the treatment, such as optimizing the duration of light exposure and ensuring good coverage of the ear canal during disease. Canine patients are unlikely to remain stationery for 20 min without anesthesia, so alternative modes of application may need to be investigated. Similarly, the presence of organic matter is likely to limit transmission of the blue light and therefore affect the effectiveness of the treatment. However, cleaning and reducing discharge are common elements of otic therapy in dogs therefore it is unlikely that aBL would be used without such measures.

The variability in response of *Pseudomonas* strains to different wavelengths of aBL may reflect the diversity of the strains used. The UK isolates were drawn from three different MLST STs, while the Danish strains were from unknown STs, suggesting the strains were not closely related. This was supported by the phylogenetic analysis using whole genome sequences, which showed these strains were distributed across the tree.

The majority (5/6) of the clinical strains in this study were insensitive to EFX, demonstrated by the MIC and disk diffusion results. *P. aeruginosa* and other non-fermenting Gram-negative bacteria exhibit a low intrinsic susceptibility to fluoroquinolones, possibly due to low membrane permeability or constitutive expression of efflux pumps, when compared to other Gram-negative bacteria such as *Escherichia coli* (Fàbrega et al., 2009). Fluoroquinolone resistance in *Pseudomonas* is commonly associated with mutations in the *gyrA* (DNA gyrase subunit A), *gyrB* (DNA gyrase subunit B), *parC* (DNA topoisomerase IV subunit A), *parE* (DNA topoisomerase IV subunit B) and *mexR* (efflux pump regulator) genes (Redgrave et al., 2014). A single mutation in *gyrA* is sufficient to confer clinically important levels of resistance to fluoroquinolones (Yoshida et al., 1990; Kureishi et al., 1994; Fàbrega et al., 2009). In this study, the presence of GyrA Asp652Tyr and Thr847Ala were identified. While both mutations have been found in other studies, their association with resistance is yet to be determined (Cortes-Lara et al., 2021). Importantly, GyrB Ser466Phe was also found in three strains (2943, 29878-1 and 29758) that were resistant to EFX and this mutation has a previously reported association with resistance (Bruchmann et al., 2013). This suggests that aBL could be used as an alternative, additional or adjuvant therapy to treat EFX resistant, otherwise intractable *Pseudomonas* infections.

Five of the six clinical strains used in this study were characterized as strong biofilm producers, while the remaining strain produced no quantifiable biofilm. This is in line with what has been reported for other *P. aeruginosa* isolates from canine OE, with 40–60% of isolates producing detectable levels of biofilm (Pye et al., 2013, 2014; Chan et al., 2019; Robinson et al., 2019). The present study investigated the use of aBL and EFX on planktonic cells, and it is possible that biofilm production would reduce the efficacy of both treatments. The efficacy of aBL (400 nm) against pre-formed *P. aeruginosa* and other nosocomial pathogen biofilms has been assessed previously (Halstead et al., 2016). This found that *Pseudomonas* biofilm “pegs” were unable to grow when added to fresh culture media after aBL-irradiation, suggesting inactivation of the biofilm.

For some strains, the usage of aBL and EFX in combination was more effective than either treatment used alone. This suggests that the concomitant use of aBL and EFX should be explored further as it seems to be a more promising treatment option than the usage of the antimicrobial alone, which may extend the useful life of existing antimicrobial therapy. One explanation for the potentially synergistic effect seen with blue light and EFX could be attributed to superoxide production. Application of aBL results the production of superoxide anions (Leanse et al., 2022), additionally, EFX inhibits DNA gyrase which has also been shown to generate superoxide in *E. coli* (Dwyer et al., 2007). In addition, mutant escape is less likely when using two antimicrobial treatments with different mechanisms of killing. The focus of the present study was EFX because of its frequent use in cases of canine otitis externa. However, it is not the only licensed antimicrobial used for treating this condition, (Nuttall, 2016) and aBL may interact with this agents differently.

Previous studies with aBL have found no evidence of resistance development to the treatment, even after multiple cycles of exposure (Wang et al., 2017a). This was also the case with the present study, where exposure of colonies recovered from aBL-treated cultures did not result in reduced efficacy (data not shown). Interestingly, the 375 nm wavelength, which is closest to the UV spectrum, was the least effective treatment. As such, the energy provided by the radiation is clearly not the main reason behind the reduced viability of the bacterial cells given that the most effective treatments are in the 400 to 470 nm range (Wang et al., 2017b). Whilst blue light is considered relatively safe in eukaryotic cells, the risks of toxicity increase with shorter wavelengths and increasing dose, partly as a consequence of thermal effects. Further work would be required to optimize dosing parameters (wavelength, irradiance, exposure time, doses, pulsing) for clinical translation.

The molecular mechanism underlying the antimicrobial effect of aBL may be the result of photosensitization of intracellular chromophores such as endogenous porphyrins (Wang et al., 2017a). Porphyrins, such as coproporphyrin III, are naturally expressed in some microorganisms and, after being excited by light absorption (405–420 nm), can lead to the production of reactive oxygen species which in turn have the cytotoxic effect on the cell (Dai and Hamblin, 2017). Other groups have used chemical substances, such as quinine, to enhance the effect of aBL on diverse microorganisms (Leanse et al., 2020a,b).

Antimicrobial resistance is a significant and growing issue worldwide. *Pseudomonas* is among the ESKAPEE group of pathogens which are of critical importance in human and animal infections. The availability of treatments, other than antimicrobial chemotherapy, will be a crucial part of strategies to combat AMR in the future (O’Neill, 2016). In the present study, we have demonstrated that aBL can significantly reduce the viability of *P. aeruginosa* isolates from clinical canine OE cases. The combination of aBL and EFX was found to be more effective than either treatment alone in most cases, suggesting that this could be used in recalcitrant infections, even when the bacteria are resistant to antimicrobials. Although further research on the optimization and synergy between these treatments is needed, aBL offers a promising way of augmenting, extending the life of, and in some cases offering an alternative to the use of antibiotics alone in the treatment of otherwise intractable canine otitis externa infections in dogs caused by *P. aeruginosa*.

## Data availability statement

The original contributions presented in this study are publicly available. The genome sequences derived in this study can be accessed from GenBank under the BioProject accession number PRJNA1078132.

## Author contributions

AG: Formal analysis, Investigation, Visualization, Writing – original draft, Writing – review & editing. MH: Conceptualization, Formal analysis, Investigation, Methodology, Supervision, Writing – original draft. BS: Formal analysis, Investigation, Methodology, Visualization, Writing – original draft, Writing – review & editing. SS: Supervision, Writing – review & editing. PC: Conceptualization, Data curation, Formal analysis, Funding acquisition, Investigation, Methodology, Project administration, Resources, Software, Supervision, Validation, Visualization, Writing – review & editing. WP: Conceptualization, Funding acquisition, Writing – review & editing. MM: Conceptualization, Funding acquisition, Project administration, Supervision, Writing – review & editing. RA: Conceptualization, Formal analysis, Funding acquisition, Investigation, Methodology, Project administration, Resources, Supervision, Writing – original draft, Writing – review & editing.
