# Supplementary material for: Exposure to blue light reduces antimicrobial resistant Pseudomonas aeruginosa isolated from dog ear infections
Source: Front Microbiol. 2024 Jul 4;15:1414412. doi: 10.3389/fmicb.2024.1414412 (PMC11255781; doi:10.3389/fmicb.2024.1414412)
Supplement: Supplementary file 1 [file Data_Sheet_1.pdf]

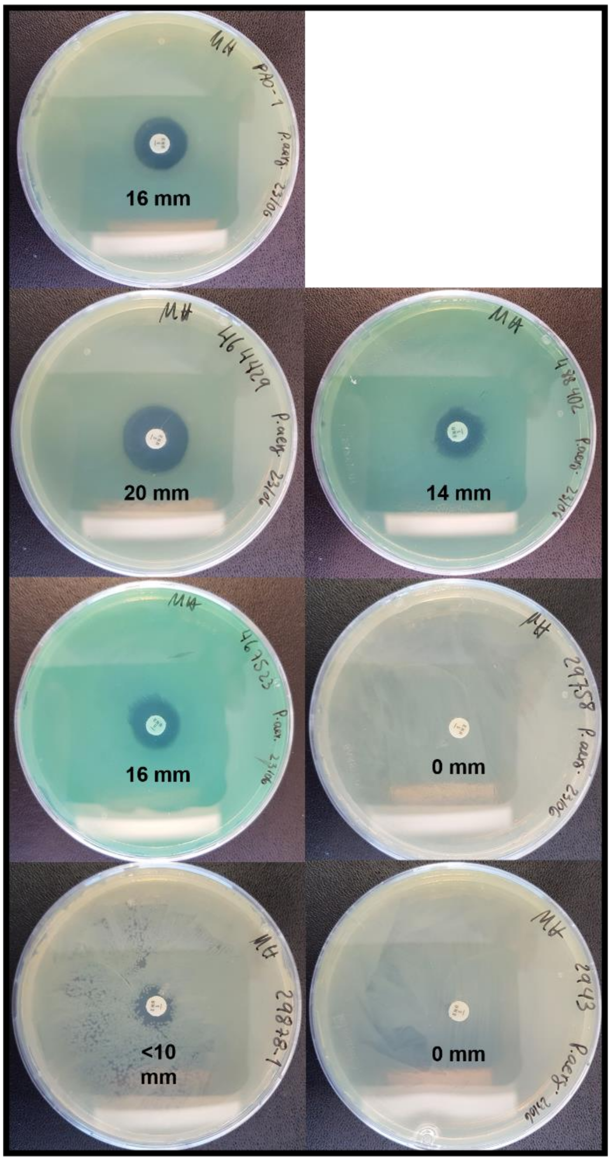

Supplementary Figure 1: Sensitivity of *Pseudomonas* isolates to enrofloxaxin.

The image depicts the seven strains used on the aBL assay, where an enrofloxacin (ENR) infused disk is placed onto a Mueller-Hinton agar plate that has been streaked with a *Pseudomonas aeruginosa*. The resulting measurement represents the distance across the clear area surrounding the disk (diameter) where bacterial growth was visibly hindered in millimetres (mm). PAO-1=16 mm; 464429= 20 mm; 467523= 16 mm; 29878-1< 10 mm; 488402= 14 mm; 297580= 0 mm; 2943= 0 mm.

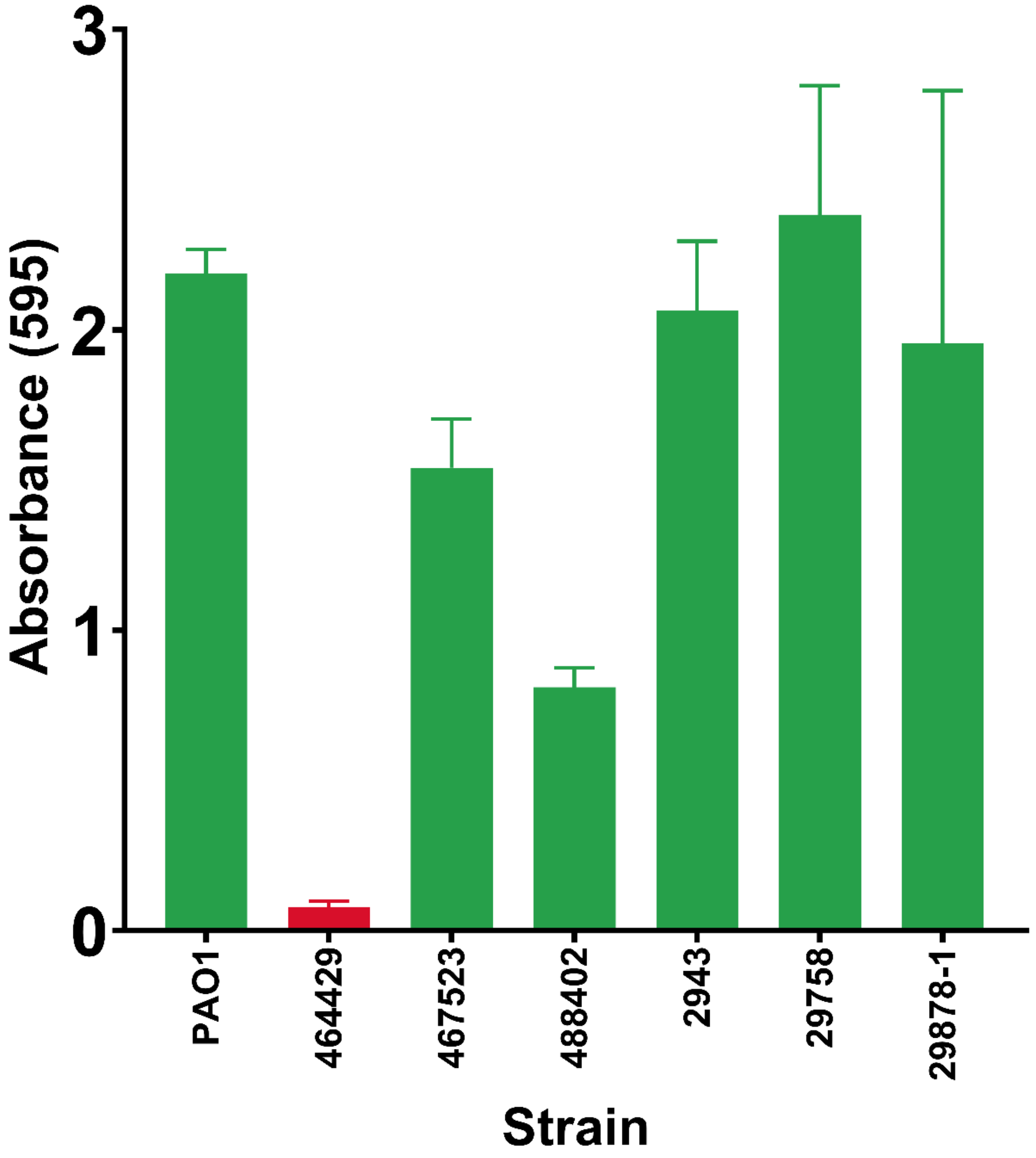

Supplementary Figure 2: Biofilm formation of clinical *P. aeruginosa* isolates in addition to PAO1. Green indicates strong biofilm production and red no quantifiable biofilm. The values shown represent three biological and six technical repeats by the mean with 95% confidence interval.

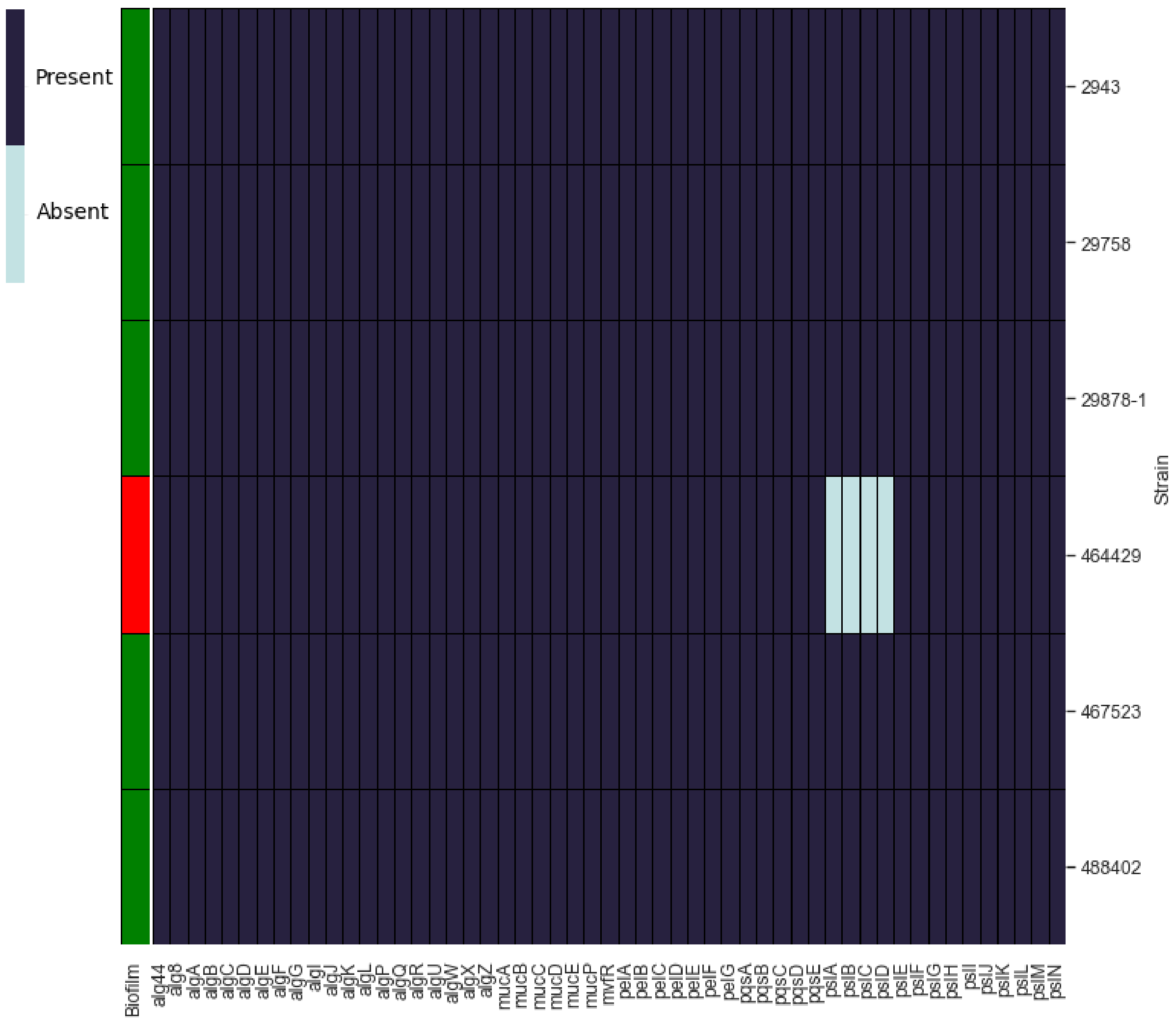

Supplementary Figure 3. Presence and absence of the sequence of 53 genes that are associated with biofilm formation from *P. aeruginosa* PAO1 in comparison to the clinical *P. aeruginosa* isolates. Additionally, the level of biofilm produced in a 96-well microtiter plate assay is shown with green indicating strong biofilm formation and red no quantifiable biofilm.

Supplemental Table 1: The average absolute irradiance of each LED channel.

Wavelength (nm)	Irradiance (mW/cm^2)
375	98 ± 17
395	366 ± 40
405	372 ± 38
420	362 ± 23
450	338 ± 94

**Gene**	**Locus Tag**	**Description**
pelA	PA3064	hypothetical protein
pelB	PA3063	pellicle/biofilm biosynthesis protein
pelC	PA3062	pellicle/biofilm biosynthesis outer membrane protein
pelD	PA3061	pellicle/biofilm biosynthesis protein
pelE	PA3060	pellicle/biofilm biosynthesis protein
pelF	PA3059	pellicle/biofilm biosynthesis glycosyltransferase
pelG	PA3058	pellicle/biofilm biosynthesis Wzx-like polysaccharide transporter
pslA	PA2231	biofilm formation protein PslA
pslB	PA2232	biofilm formation protein PslB
pslC	PA2233	biofilm formation protein PslC
pslD	PA2234	biofilm formation protein PslD

pslE	PA2235	biofilm formation protein PslE
pslF	PA2236	biofilm formation protein PslF
pslG	PA2237	biofilm formation protein PslG
pslH	PA2238	biofilm formation protein PslH
pslI	PA2239	biofilm formation protein PslI
pslJ	PA2240	biofilm formation protein PslJ
pslK	PA2241	biofilm formation protein PlsK
pslL	PA2242	biofilm formation protein PslL
pslM	PA2243	bFAD-binding dehydrogenase
pslN	PA2244	hypothetical protein
algD	PA3540	GDP-mannose 6-dehydrogenase AlgD
alg8	PA3541	glycosyltransferase alg8
alg44	PA3542	alginate biosynthesis protein Alg44

algK	PA3543	alginate biosynthesis protein AlgK
algE	PA3544	alginate production protein AlgE
algG	PA3545	alginate-c5-mannuronan-epimerase AlgG
algX	PA3546	alginate biosynthesis protein AlgX
algL	PA3547	alginate lyase
algI	PA3548	alginate o-acetylase AlgI
algJ	PA3549	alginate o-acetylase AlgJ
algF	PA3550	alginate o-acetyltransferase AlgF
algA	PA3551	bifunctional mannose-1-phosphate guanylyltransferase/mannose-6-phosphate isomerase
mvfR	PA1003	transcriptional regulator MvfR
pqsA	PA0996	anthranilate--CoA ligase
pqsB	PA0997	hypothetical protein
pqsC	PA0998	hypothetical protein
pqsD	PA0999	3-oxoacyl-ACP synthase
pqsE	PA1000	thioesterase PqsE

algU	PA0762	RNA polymerase sigma factor AlgU
mucA	PA0763	sigma factor AlgU negative regulator MucA
mucB	PA0764	sigma factor AlgU regulator MucB
mucC	PA0765	positive regulator for alginate biosynthesis MucC
mucD	PA0765	serine protease MucD
mucP	PA3649	metalloprotease protease
mucE	PA4033	small envelope protein MucE
algW	PA4446	AlgW protein
algP	PA5253	alginate regulatory protein AlgP
algQ	PA5255	anti-RNA polymerase sigma 70 factor
algR	PA5261	alginate biosynthesis regulatory protein AlgR
algZ	PA5262	alginate biosynthesis protein AlgZ/FimS
algC	PA5322	phosphomannomutase
algB	PA5483	two-component response regulator AlgB

Supplementary Table 2: List of genes associated with biofilm formation that were investigated

Strain	MIC (mg/L)
PAO-1	2
2943	>32
29758	>32
29878-1	>32
464429	1
467523	4
488402	2

Supplementary Table 3: *Pseudomonas aeruginosa* isolates minimum inhibitory concentration (MIC) when using EFX in micro-dilution method.
